# Non-Invasive Cardiac Output Measurement in Low and Very Low Birth Weight Infants: A Method Comparison

**DOI:** 10.3389/fped.2014.00016

**Published:** 2014-03-25

**Authors:** Oswin Grollmuss, Patricia Gonzalez

**Affiliations:** ^1^Centre Chirurgical Marie Lannelongue, INSERM 999, Université Paris XI Sud, Orsay, France; ^2^Institut de Puériculture et de Périnatalogie, Université Paris V Descartes, Clamart, France

**Keywords:** low birth weight infants, very low birth weight infants, neonatal intensive care unit, pediatric cardiac intensive care unit, stroke volume, cardiac output, electrical velocimetry, transthoracic echocardiograph

## Abstract

**Background:** Cardiac output (CO) measurement in low (LBW) and very low (VLBW) birth weight infants is difficult. Hitherto, sporadical transthoracic echocardiography (TTE) is the only non-invasive measurement method. Electrical velocimetry (EV) has been evaluated as an alternative in normal weight newborns.

**Objectives:** The study was designed to evaluate if EV could be interchangeable with TTE even in LBW and VLBW infants.

**Methods:** In 28 (17 LBW, 11 VLBW) pre-mature newborns, *n* = 228 simultaneous TTE (trans-aortic Doppler), and EV measurements (134 LBW, 94 VLBW) of stroke volume (SV) and heart rate (HR) were performed, thereof calculating body weight indexed SV (=SV*) and CO (=CO*) for all patients and the subgroups. Method comparison was performed by Bland–Altman plot, method precision expressed by calculation of the coefficient of variation (CV).

**Results:** Mean CO* in all patients was 256.4 ± 44.8 (TTE) and 265.3 ± 48.8 (EV) ml/kg/min. Bias and precision were clinically acceptable, limits of agreement within the 30% criterion for method interchangeability (17). According to their different anatomic dimensions and pathophysiology, there were significant differences of SV(*), HR, and CO* for LBW and VLBW infants as well for inotropic treatment and ventilation mode.

**Conclusion:** Extending recent publications on EV/TTE comparison in newborns, this study suggests that EV is also applicable in LWB/VLBW infants as a safe and easy to handle method for continuous CO monitoring in the NICU and PCICU.

## Introduction

Information about cardiac output (CO) in newborn and especially in pre-term, low birth weight (LBW), and very low birth weight (VLBW) infants is difficult to obtain. However, this information is particularly important in such patients with their numerous circulatory features [heart–lung interaction (1, 2), sepsis (3), changes in circulatory volume, need for catecholamine treatment, hemorrhagy].

Clinical observation does not deliver sufficient information (4). Invasive (potentially traumatic) and non-invasive blood pressure measurement (5), influenced by vascular resistances, reflects CO only partially. Electrocardiogram (ECG) and heart rate (HR) variability only give indirect information. NIRS indicates compromised regional circulation possibly related to an underlying general low flow (6) but can deliver no evidence on its cause.

In LBW infants, conventional invasive CO measurement methods, often considered as “gold standards” like the Swan – Ganz catheter, are too traumatic in these patients, therefore research for non-invasive alternatives is necessary. Transthoracic cardiac ultrasound (TTE) with trans-aortic Doppler (7) is used in daily clinical practice, but is technically highly demanding and can only be applied sporadically. Another possible technique, bioimpedance, referring to thoracic impedance changes by CO-dependent intrathoracic blood flow, has been developed in the 60s by Kubicek (8). One of its most recent modifications is electrical velocimetry (EV) based on the mathematical algorithms formulated by Bernstein and Lemmens (9) in 2005.

Electrical velocimetry and trans-aortic Doppler have been validated against invasive CO measurement reference techniques [thermodilution (10, 11), Fick (12)]. EV has been evaluated against the Fick and the thermodilution method as gold standards, but data are scarce, the cohorts enrolled in the studies heterogenous, and the results remain controversial concerning the measurement of “true” CO. On the other hand, with special regard to clinical purposes, EV has been found interchangeable with Doppler ultrasound in adults (13) and, recently, evidence was given for the interchangeability of the two methods in newborns with and without underlying congenital heart disease (14–16).

The aim of this method comparison study was to investigate if EV and TTE are even interchangeable in LBW and VLBW infants in order to create a rationale for further clinical validation studies of EV.

## Methods

In this prospective, observational study, we performed 228 CO measurements in 28 pre-term newborns (subgroups: 17 LBW, 11 VLBW). Detailed epidemiological data are given in Table 1.

**Table 1:** **Epidemiological data of the patients enrolled in the study**.

Parameter	Total
Number of patients (measurements)	28 (228)
Number of LBW infants (measurements)	17 (134)
Number of VLBW infants (measurements)	11 (94)
Male (patients)	18
Female (patients)	10
Mean gestational age (weeks), all patients	31.7 ± 3.1
Mean gestational age (weeks), VLBW	29.2 ± 2.8
Median age at exam (days)	15 (1 − 48)
Mean weight at exam (kg), all patients	1.618 ± 0.346
Mean weight at exam (kg), LBW	1.866 ± 0.145
Mean weight at exam (kg), VLBW	1.236 ± 0.161
Patients ventilated (number)	19
Inotropic support (patients)	10

The study was designed as an observational method comparison study for the interchangeability of TTE and EV and not as a clinical validation study. Nevertheless, clinical observations will be communicated for correlation of SV and CO measurements with body weight, and observations for SV and CO under inotropic treatment (epinephrine 0.05 μg/kg/min and milrinone 0.375 μg/kg/min) and under different respiration conditions that may illustrate the utility of EV (and TTE) in daily clinical practice.

The local ethical committee’s permission and informed parental consent were obtained in accordance with the ethical standards laid down in the 1964 Declaration of Helsinki and its later amendments.

### Electrical velocimetry

Stroke volume (SV) was measured by EV as SV_EV_ using the ICON^®^ bioimpedance monitor (Osypka Medical, La Jolla, CA, USA). The principles of EV and the method itself have been discussed elsewhere in detail (9, 16). CO_EV_ was measured as:

$$CO_{\mathrm{EV}}=SV_{\mathrm{EV}}\times \mathrm{HR}\;\left(\mathrm{ml}∕\min\;\right)$$

and indexed to body weight as ${\mathrm{CO}}_{\mathrm{EV}}^{*}$ (ml/kg/min), SV_EV_ indexed to body weight as ${\mathrm{SV}}_{\mathrm{EV}}^{*}\;(\mathrm{ml∕kg)}$.

**Figure 1:**
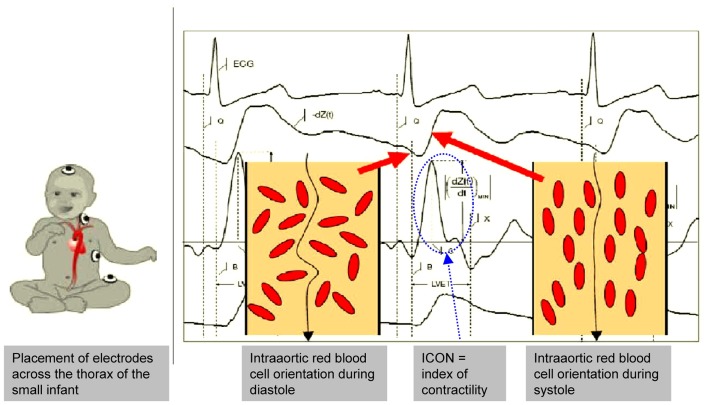
**EV – electrode placement in the small infant and EV signals**. It is important that the electrode placing keeps sufficient distance between the electrodes to avoid interferences and signal disturbances which may be difficult in very small infants. ECG and EV signals must be clearly identified on the monitor as they correlate with the intra-aortic blood flow changes. Further explanations are given in the text. (With kind permission of Osypka Medical, Berlin, Germany and La Jolla, CA, USA, modified for scientific publication by the authors).

Utmost attention was paid to the best signal quality and ECG and d*Z*/d*t* curve on the ICON^®^ monitor (Figure 1). Conventional pediatric electrodes were placed according to the recommendations for the use of the ICON^®^ in small children (Figure 1). EV (SV_EV_) and trans-aortic Doppler measurements of SV (SV_TTE_) were performed simultaneously.

### Trans-aortic Doppler ultrasound

SV_TTE_ measurements were performed as described in detail by Grollmuss et al. (16). For the measurement of the velocity time integral (VTI), utmost attention was paid to the ultrasound sample being placed directly behind the aortic valve, in line with the blood stream ejected from the left ventricle into the aorta. CO_TTE_ and the body weight indexed ${\mathrm{CO}}_{\mathrm{TTE}}^{*}\;\left(\mathrm{ml∕kg∕min}\right)$ were then calculated in analogy to the EV measurements. SV_TTE_ was indexed to body weight as ${\mathrm{SV}}_{\mathrm{TTE}}^{*}\;\left(\mathrm{ml∕kg}\right).$

In order to minimize artifacts end errors of the EV and TTE measurements that have been described in the literature, in particular referring to the exact measurement of the diameter of the aortic annulus five subsequent measurements were made under optimal measurement and signal conditions and then averaged.

### Statistical methods

As neither TTE nor EV represent a gold standard for CO measurement, they were considered as interchangeable when the limits of agreement for CO measured by the two methods fulfilled the criteria (30% of mean) formulated by Critchley and Critchley (17).

SigmaPlot software, version 12.3 (Systat Software, Chicago, IL, USA) was used for method comparison (Bland–Altman Plot), descriptive statistics, group comparison (*t*-test), and correlation tests (Pearson) (17–19). Differences between the values were considered as significant at *p* < 0.05, if not indicated otherwise. Values are given as means ± SD, if not indicated otherwise. The coefficient of variation (CV) was calculated as the expression of the method’s precision.

The Doppler SV_TTE_ measurements were performed by a certified and experienced specialist in neonatal cardiac ultrasound, who was blind for the simultaneous EV measurements using the ATL hdi 5000 ultrasound machine, now Philips, Andover, MA, USA and the Vivid I and Vivid 7 ultrasound machines, both from GE healthcare, Chalfont St. Giles, Buckinghamshire, UK.

## Results

### Patients’ data

Mean gestational age of all patients was 31.7 ± 3.1 weeks (VLBW 29.2 ± 2.8 weeks), 27/28 (=96.4%) being pre-term born infants with 24/28 (=85.7%) being under 35 and 8/28 (=28.6%) under 30 weeks of gestation (minimal gestational age: 26 weeks). Mean weight of all patients at the time of CO measurement was 1.618 ± 0.346 kg, mean weight of the VLBW patients 1.236 ± 0.161 kg, the smallest infant enrolled in the study weighing 0.860 kg. Ten out of 28 patients (35.7%) had low dose inotropic support, 19/28 patients (=67.9%) were artificially ventilated.

### Method comparison

**Figure 2:**
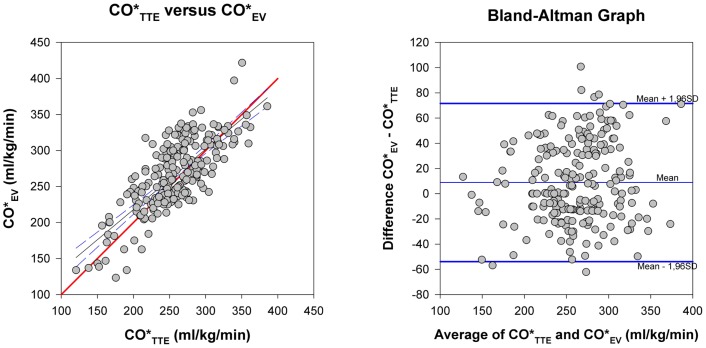
**Method comparison**. Left: CO*_TTE_ versus CO*_EV_. The thick (red) line is the line of identity. Right: Bland-Altman plot for differences of CO*_EV_−CO*_TTE_ against the average of the two methods. Thick (blue) lines: limits of agreement, thin (blue) line: mean of the methods. Its distance to the zero line is the bias of the two methods.

Bland–Altman method comparison for ${\mathrm{CO}}_{\mathrm{TTE}}^{*}$ and ${\mathrm{CO}}_{\mathrm{EV}}^{*}$ and precision calculation are given in Table 2 and Figure 2. Bias was consistently positive for all patients and the subgroups with consistently higher ${\mathrm{CO}}_{\mathrm{EV}}^{*}$ than ${\mathrm{CO}}_{\mathrm{TTE}}^{*}$ values and clinically acceptable with values <10% of the mean of both methods. Limits of agreement for both methods in all patients and the subgroups were below the 30% limits stipulated by Critchley and Critchley (17) for method interchangeability (Figure 2). There was practically no difference of EV and TTE interchangeability for CO measurement between ventilated and non-ventilated infants with bias of 4.0% of mean in ventilated patients and 2.5% in non-ventilated patients and limits of agreement of 23.6 and 25.2% for ventilated and non-ventilated infants, respectively.

**Table 2:** **Bland–Altman test for method agreement and coefficient of variations (CV) for precision calculation in all patients, LBW and VLBW infants**.

Population	Method	Mean	CV (%)	Agreement (Bland–Altman)				
				Upper limit	Lower limit	% of mean	Bias	% of mean
All patients	TTE	256.4 ± 44.8	8.0	71.6	−53.8	24.0	8.9	3.4
	EV	265.3 ± 48.8	6.3	
LBW infants	TTE	248.3 ± 39.9	8.7	70.9	−50.2	23.9	10.4	4.1
	EV	258.7 ± 44.7	7.1	
VLBW infants	TTE	274.2 ± 53.8	10.5	69.1	−48.4	23.5	5.3	1.9
	EV	268.8 ± 49.0	7.0	

*Means, bias, and limits are expressed as absolute values (ml/kg/min), bias and limits also as % of means*.

### Precision of the methods

Overall CV was <10%, expressing clinically acceptable precision of TTE [in accordance with Hudson et al. (7)] and EV [in accordance with Grollmuss et al. (16)]. Whereas CV for EV was nearly identical in all groups, CV for TTE was significantly higher in the VLBW than in the LBW group (*p* = 0.009), indicating that CO measurement by trans-aortic Doppler may be technically more difficult in the smallest infants. CV for EV in all patients was significantly lower than for TTE (*p* = 0.046) suggesting that EV may be somewhat more precise for CO measurement in small infants than TTE. There was a significantly higher CV for ${\mathrm{CO}}_{\mathrm{EV}}^{*}$ in spontaneously breathing (mean 11.2 ± 8.5%) than in ventilated infants (mean 8.2 ± 9.4%, *p* = 0.025), thus less precision for EV in extubated patients.

### Descriptive statistics

Descriptive statistics for SV_TTE_, SV_EV_, ${\mathrm{SV}}_{\mathrm{TTE}}^{*},$ ${\mathrm{SV}}_{\mathrm{EV}}^{*},$ ${\mathrm{CO}}_{\mathrm{TTE}}^{*},$ ${\mathrm{CO}}_{\mathrm{EV}}^{*}$, and HR are given in Table 3.

**Table 3:** Circulatory parameters: Measured SV, body weight indexed SV*, HR, and body weight indexed CO* for all patients, LBW and VLBW.

Parameter population	SV (ml)		SV* (ml/kg)		HR (beats/min)	CO* (ml/kg/min)	
	TTE	EV	TTE	EV	ECG (EV)	TTE	EV
All patients	2.97 ± 0.68	3.04 ± 0.75	1.63 ± 0.27	1.68 ± 0.28	157.7 ± 14.6	256.4 ± 44.8	265.3 ± 48.8
LBW	3.31 ± 0.53	3.46 ± 0.56	1.62 ± 0.25	1.69 ± 0.25	158.2 ± 10.8	247.7 ± 39.8	259.1 ± 44.1
VLBW	2.49 ± 0.58	2.45 ± 0.58	1.65 ± 0.29	1.68 ± 0.31	163.5 ± 12.3	268.8 ± 49.0	274.2 ± 53.8

*Measurements performed by TTE and EV*.

#### Stroke volume

As could be expected, SV_TTE_ and SV_EV_ were significantly lower in VLBW patients compared to those in the LBW group due to their smaller anatomic dimensions (*p* < 0.001) whereas there was no statistically significant difference between VLBW and LBW infants for ${\mathrm{SV}}_{\mathrm{TTE}}^{*}$ and ${\mathrm{SV}}_{\mathrm{EV}}^{*}$. Correlation between SV_TTE_ and body weight was *r* = 0.77 and between SV_EV_ and body weight *r* = 0.81.

#### Cardiac output

${\mathrm{CO}}_{\mathrm{TTE}}^{*}$and ${\mathrm{CO}}_{\mathrm{EV}}^{*}$ were higher in the VLBW than in the LBW group (*p* = 0.009 for ${\mathrm{CO}}_{\mathrm{TTE}}^{*}$ and 0.002 for ${\mathrm{CO}}_{\mathrm{EV}}^{*}$) due to a significantly higher HR in the VLBW group compared to the LBW patients (*p* < 0.001).

#### Inotropic treatment

SV and SV* were higher in patients with inotropic treatment (SV_EV_ 2.88 ± 0.83 ml, ${\mathrm{SV}}_{\mathrm{EV}}^{*}$ 1.73 ± 0.24 ml) than in those without catecholamine treatment (SV_EV_ 2.51 ± 0.41 ml, *p* = 0.002, ${\mathrm{SV}}_{\mathrm{EV}}^{*}$ 1.65 ± 0.29 ml, *p* = 0.031). HR was significantly in patients without (163.5 ± 9.1 beats/min) than with inotropic treatment (159.5 ± 12.2 beats/min, *p* = 0.044). Consequently, CO* was significantly higher in these patients, too (${\mathrm{CO}}_{\mathrm{EV}}^{*}$ 275 ± 40.9 vs. 258.6 ± 52.3 ml/kg/min, *p* = 0.009).

#### Ventilation effects

For all patients, ${\mathrm{CO}}_{\mathrm{EV}}^{*}$ (270.9 ± 47.6 ml/kg/min) in ventilated infants was higher than in non-ventilated patients (${\mathrm{CO}}_{\mathrm{EV}}^{*}$ 252.5 ± 49.3 ml/kg/min, *p* = 0.024) based on a higher SV_EV_ (2.77 ± 0.70 ml, ${\mathrm{SV}}_{\mathrm{EV}}^{*}$ 1.71 ± 0.25 ml) in the ventilated group compared to the non-ventilated patients (SV_EV_ 2.55 ± 0.72 ml, ${\mathrm{SV}}_{\mathrm{EV}}^{*}$ 1.63 ± 0.32, *p* = 0.031).

## Discussion

This study was a pure observational method comparison of EV and TTE in LBW and VLBW infants with supplementary clinical observations indicating a possible use of EV in daily neonatological and cardiological practice. It was not designed to evaluate a “true” CO comparing EV to a convened gold standard like Fick’s method or thermodilution, which is not possible in very small infants, but to explore the utility of EV as an easily feasible alternative to the technically demanding trans-aortic Doppler for the measurement of CO in these patients.

Method comparison of the two non-invasive CO measurement methods, TTE and EV, shows that they are interchangeable, fulfilling the criteria of Critchley and Critchley (17), with narrow limits of agreement, low and constant bias, and, finally, good precision of either method with EV being somewhat more precise than TTE.

Method interchangeability was not altered by the respiratory mode (ventilation or spontaneous respiration) whereas ${\mathrm{CO}}_{\mathrm{EV}}^{*}$ measurements were significantly less precise in extubated than in ventilated infants suggesting a particularly careful interpretation of EV in these patients as movements of the awake-patients might interfere with the EV signal.

Stroke volume correlated well with the patients’ body weight and was therefore higher in LBW than in VLBW patients, thus reflecting correctly the patients’ differences of weight and age. As the body weight related SV* was not different in the two groups, the paradoxically higher CO* in the VLBW group was obviously due to a higher HR in these patients.

The higher SV and CO in ventilated patients may be due to positive effects of positive intrathoracic pressure on left ventricular (LV) afterload reduction as has been previously reported (1, 2).

Extending the previous studies in normal weight newborns (15, 16), the present study shows that EV and TTE are also interchangeable even in LBW and VLBW infants, fulfilling the criteria of Critchley and Critchley (17) for method comparison of two non-reference methods, with clinically acceptable precision of CO measurement by EV (16).

As a conclusion, EV may represent a valuable tool for CO monitoring in LBW and VLBW infants, particularly helpful in detecting imminent risks of low CO (16) associated to the pathology of very small and pre-term newborns like circulatory degradation in the case of sepsis or bleeding, and it may help to conduct volume substitution treatment. These aspects of a possible role of EV in the critical care management of very small NICU or PCICU patients should be evaluated by further, clinical studies.

Compared to TTE, the advantage of EV may be a continuous and relatively easy manageable CO monitoring completing the “traditional” circulatory monitoring in neonatal, pediatric, and pediatric cardiological intensive care units. Its clinical utility still needs to be confirmed, but evidence is growing (15, 16). In fact, the clinical observations of the present study with regard to variations of SV_EV_, ${\mathrm{SV}}_{\mathrm{EV}}^{*}$, and ${\mathrm{CO}}_{\mathrm{EV}}^{*}$ related to weight, ventilation, and inotropic treatment support a potential role of EV in the clinical practice of CO monitoring even in very small infants.

## Conflict of Interest Statement

The Review Editor Yves Durandy declares that, despite being affiliated to the same institution as author Oswin Grollmuss, the review process was handled objectively and no conflict of interest exists. The other co-author reports no conflicts of interest.
